# Supplementary material for: External Load Analysis in Beach Handball Using a Local Positioning System and Inertial Measurement Units
Source: Sensors (Basel). 2022 Apr 14;22(8):3011. doi: 10.3390/s22083011 (PMC9026435; doi:10.3390/s22083011)
Supplement: Supplementary file 1 [file sensors-22-03011-s001.zip › sensors-1679162-supplementary.pdf]

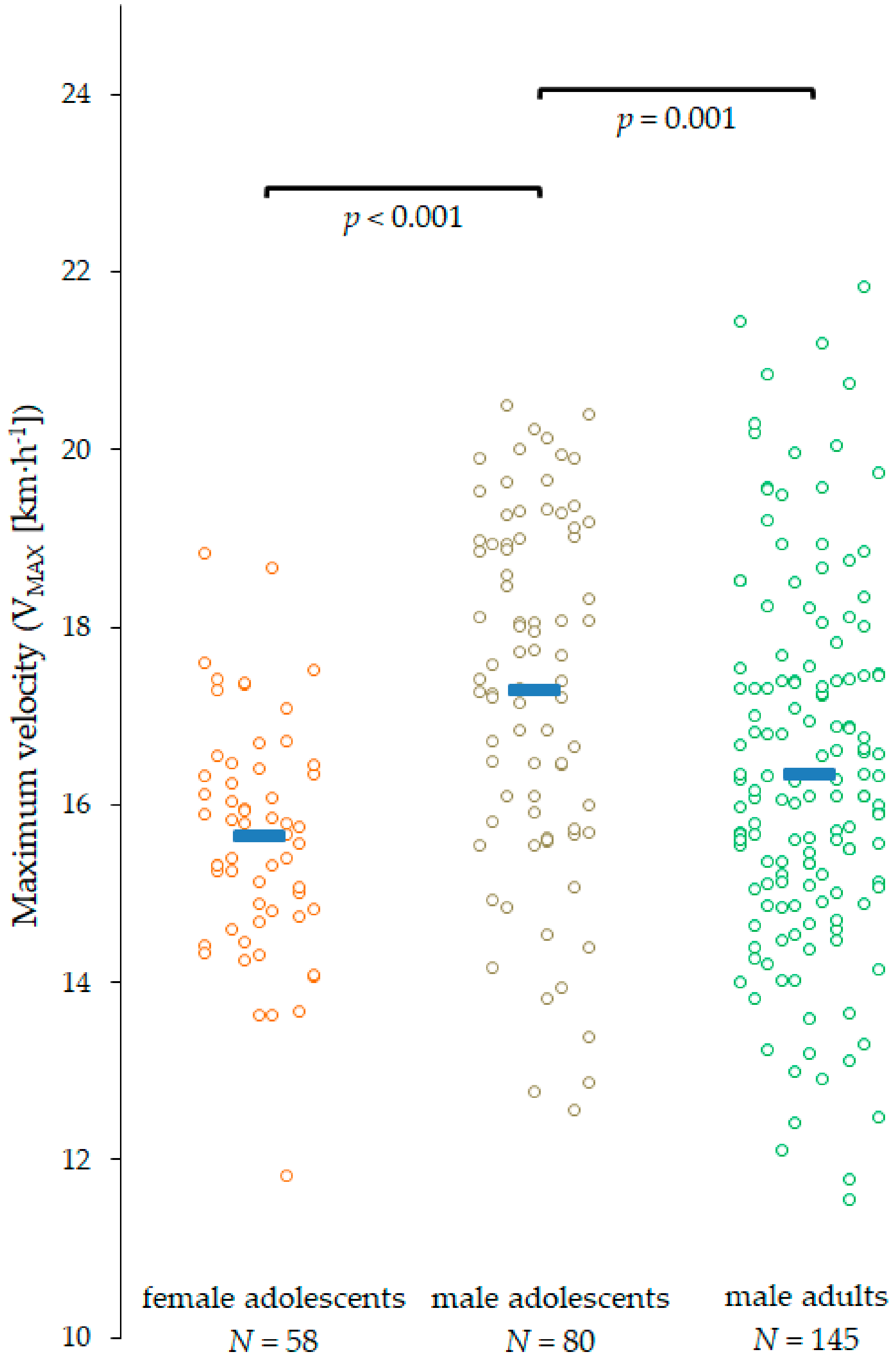

24
22
20
18
16
14
12
10
Maximum velocity (V_{MAX} [km·h^{-1}])
p < 0.001
p = 0.001
female adolescents
N = 58
male adolescents
N = 80
male adults
N = 145

(**a**)

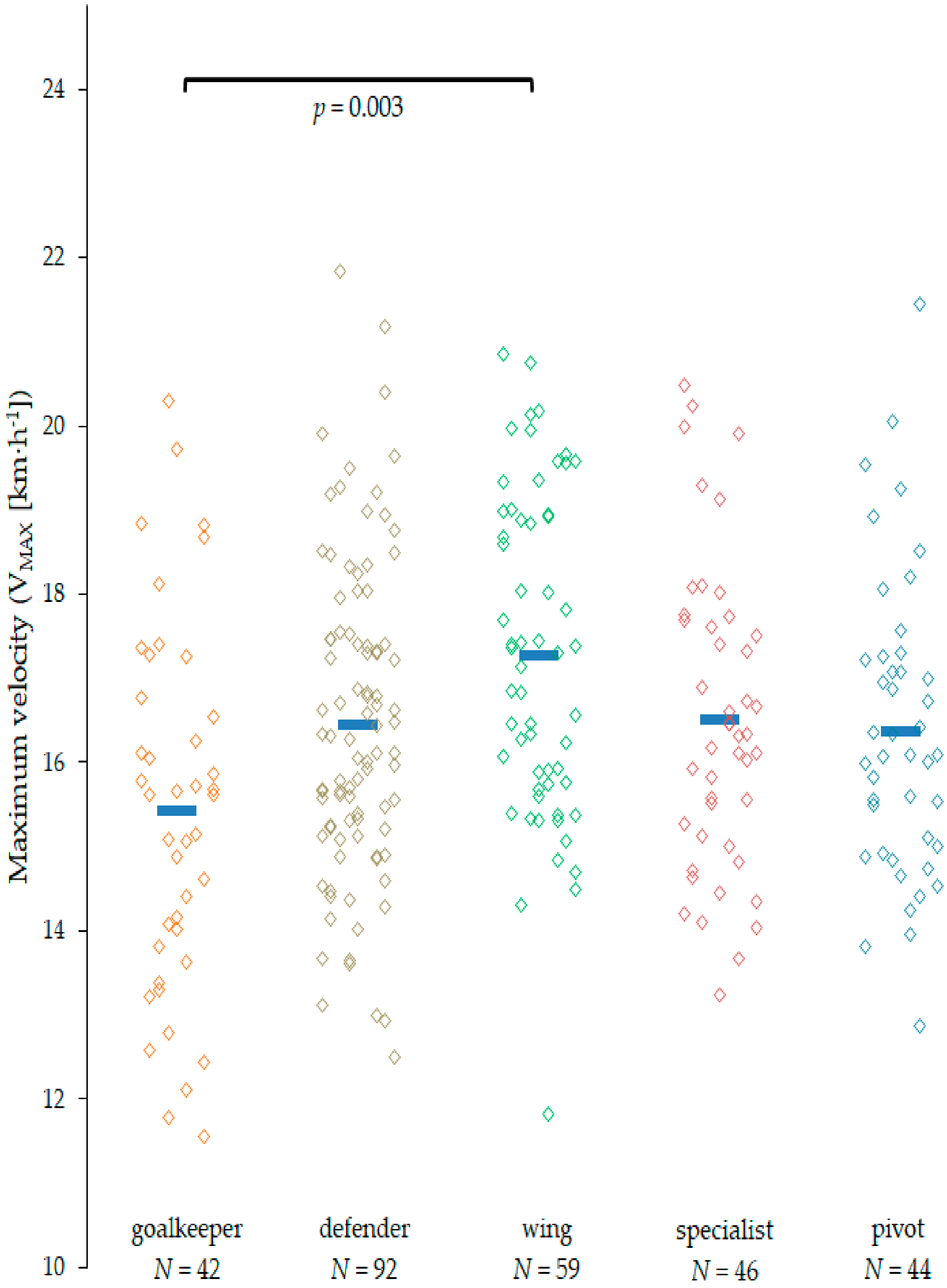

(**b**)

Figure S1. Maximum velocity (V_{MAX} [km·h^{-1}]) across the subgroups: (**a**) subgroup 1 (age/sex); (**b**) subgroup 2 (playing position).

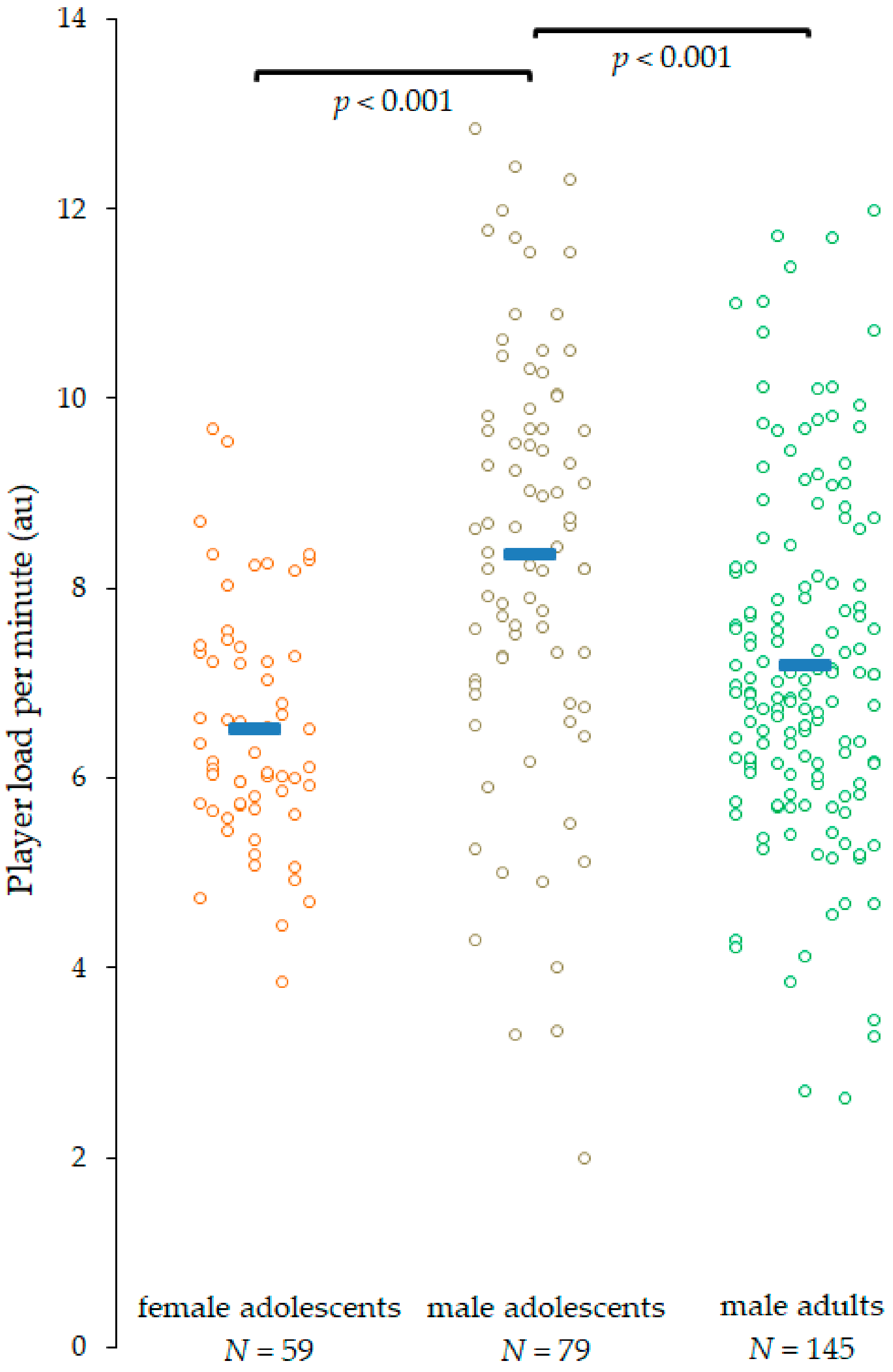

14
12
10
8
6
4
2
0
Player load per minute (au)
p < 0.001
p < 0.001
female adolescents
N = 59
male adolescents
N = 79
male adults
N = 145

(**a**)

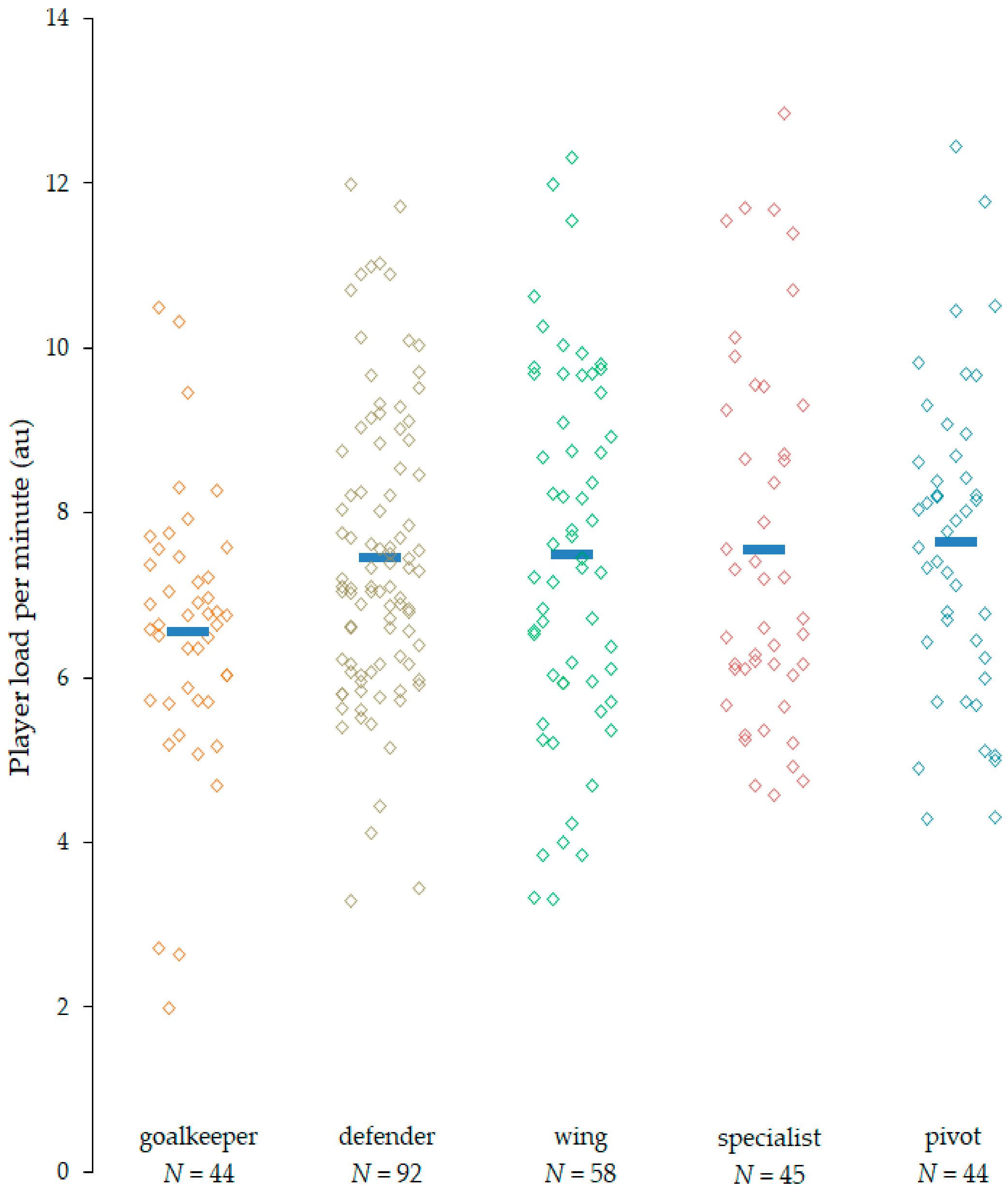

(**b**)

Figure S2. Player load per minute (arbitrary unit, au) across the subgroups: (**a**) subgroup 1 (age/sex); (**b**) subgroup 2 (playing position).

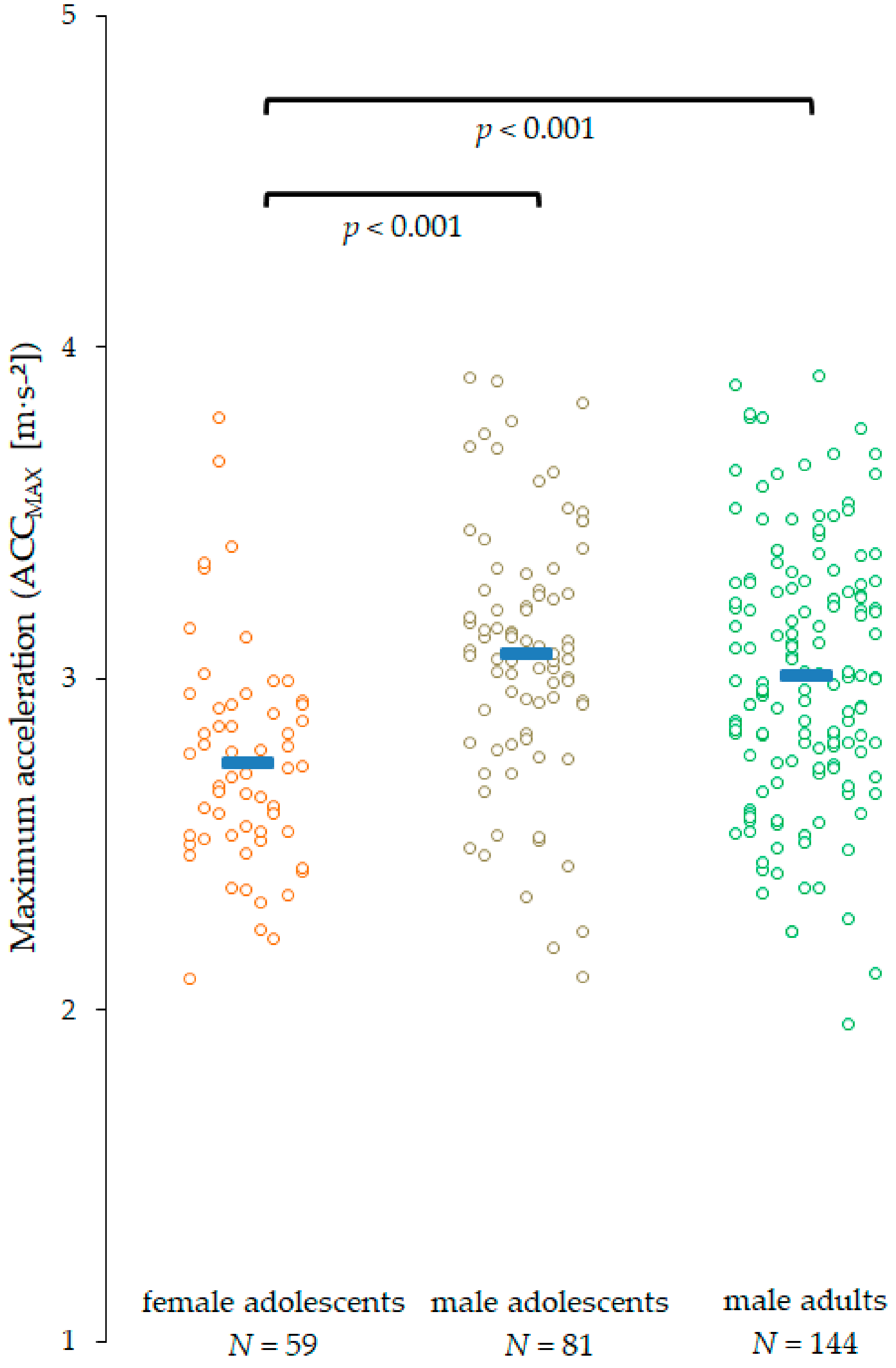

Maximum acceleration (ACC$_{MAX}$ [m·s^{-2}])
5
4
3
2
1
$p < 0.001$
$p < 0.001$
female adolescents
$N = 59$
male adolescents
$N = 81$
male adults
$N = 144$

(a)

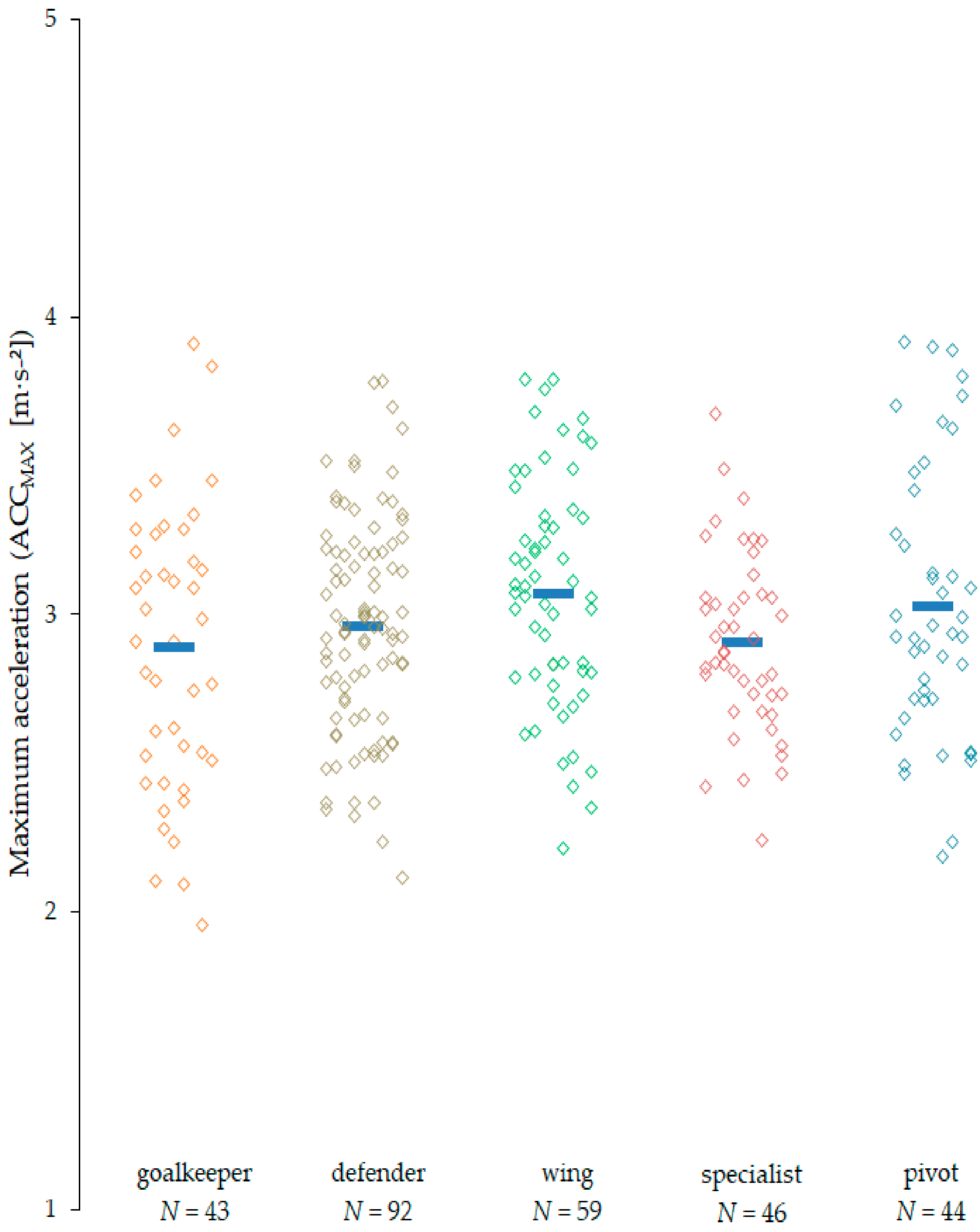

(**b**)

Figure S3. Maximum acceleration (ACC$_{MAX}$ [m·s^{-2}]) across the subgroups: (**a**) subgroup 1 (age/sex); (**b**) subgroup 2 (playing position).

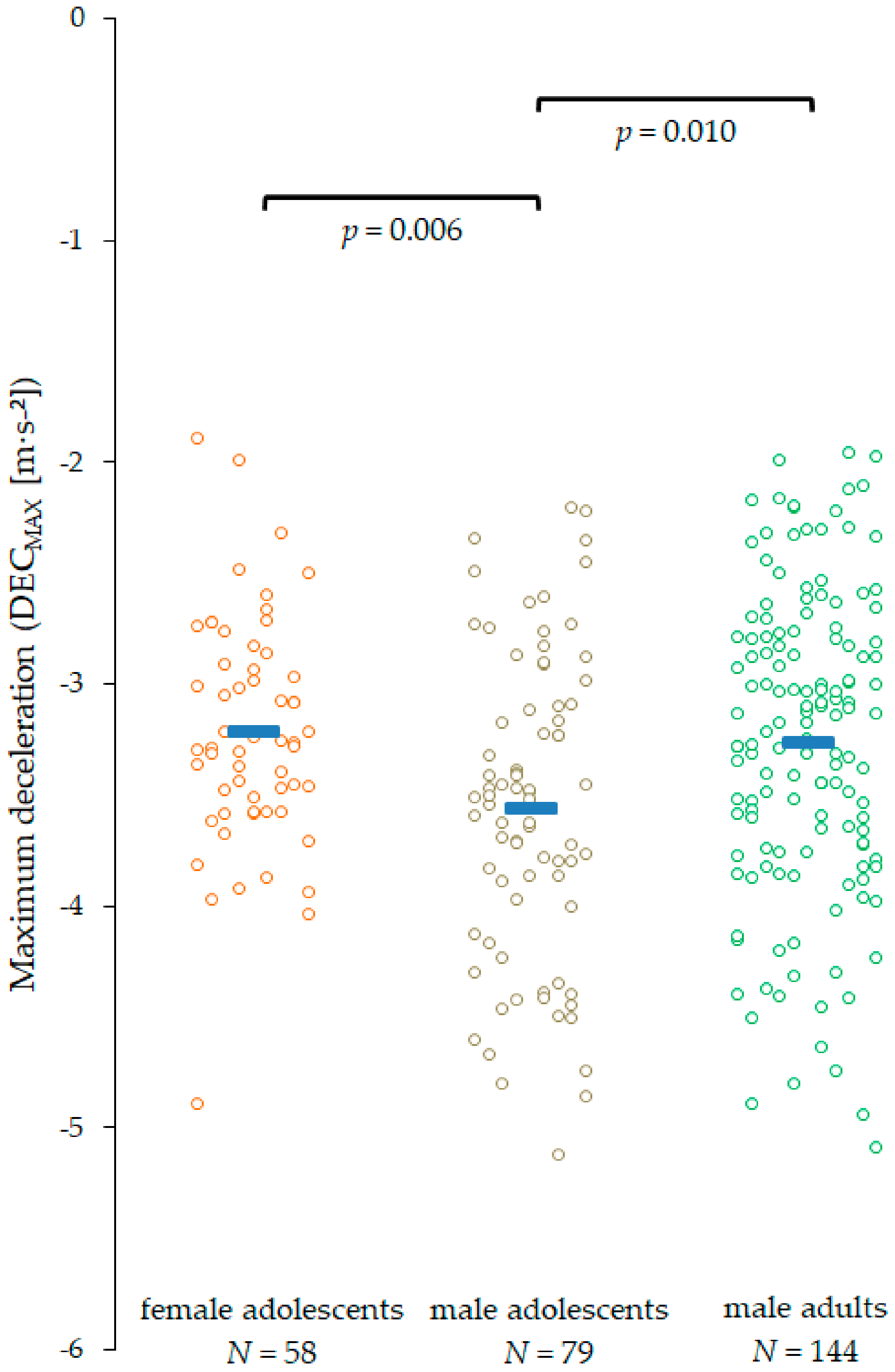

0
-1
-2
-3
-4
-5
-6
Maximum deceleration (DEC$_{MAX}$ [m·s^{-2}])
p = 0.006
p = 0.010
female adolescents
N = 58
male adolescents
N = 79
male adults
N = 144

(**a**)

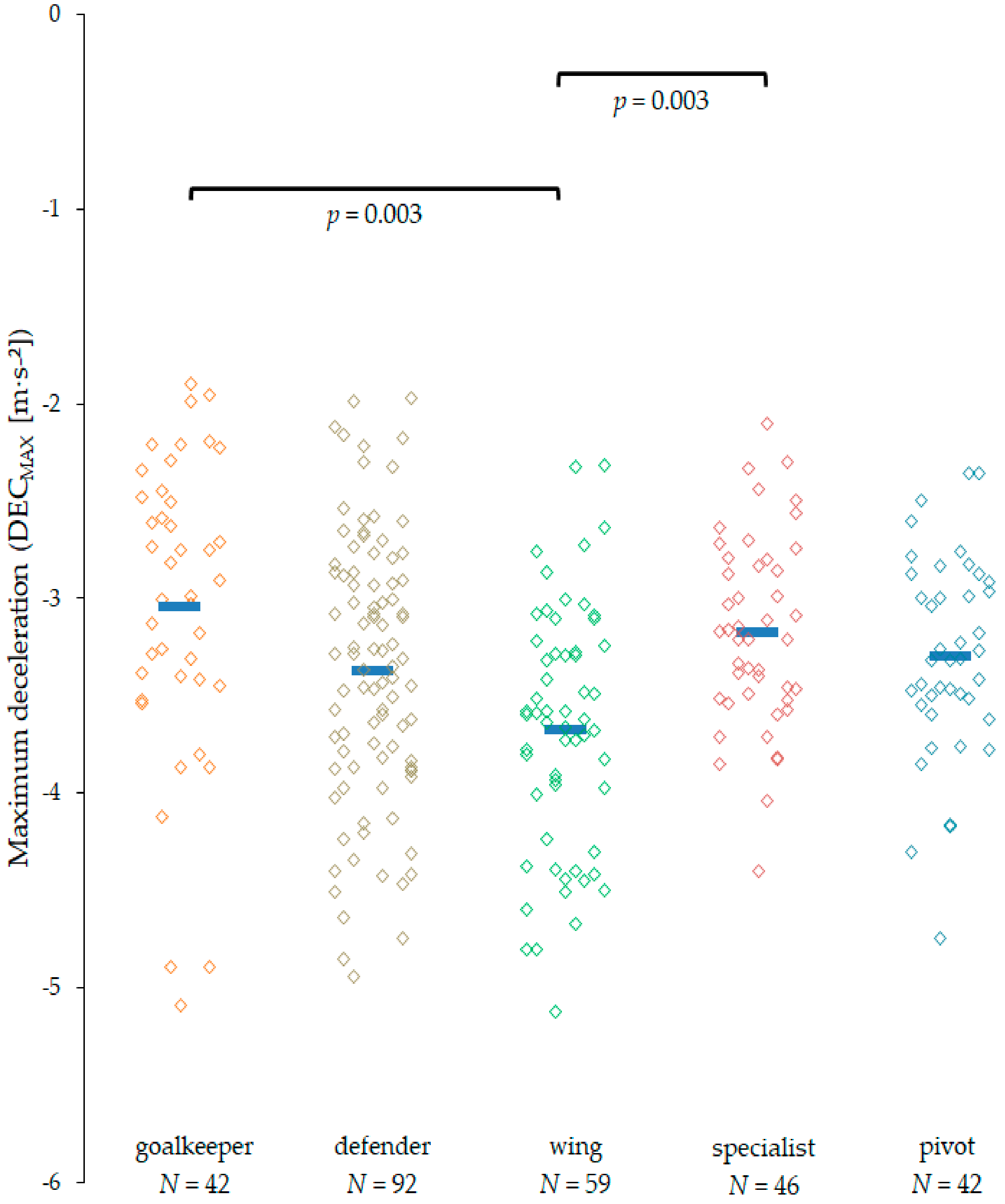

(**b**)

Figure S4. Maximum deceleration (DEC_{MAX} [$m \cdot s^{-2}$]) across the subgroups: (**a**) subgroup 1 (age/sex); (**b**) subgroup 2 (playing position).

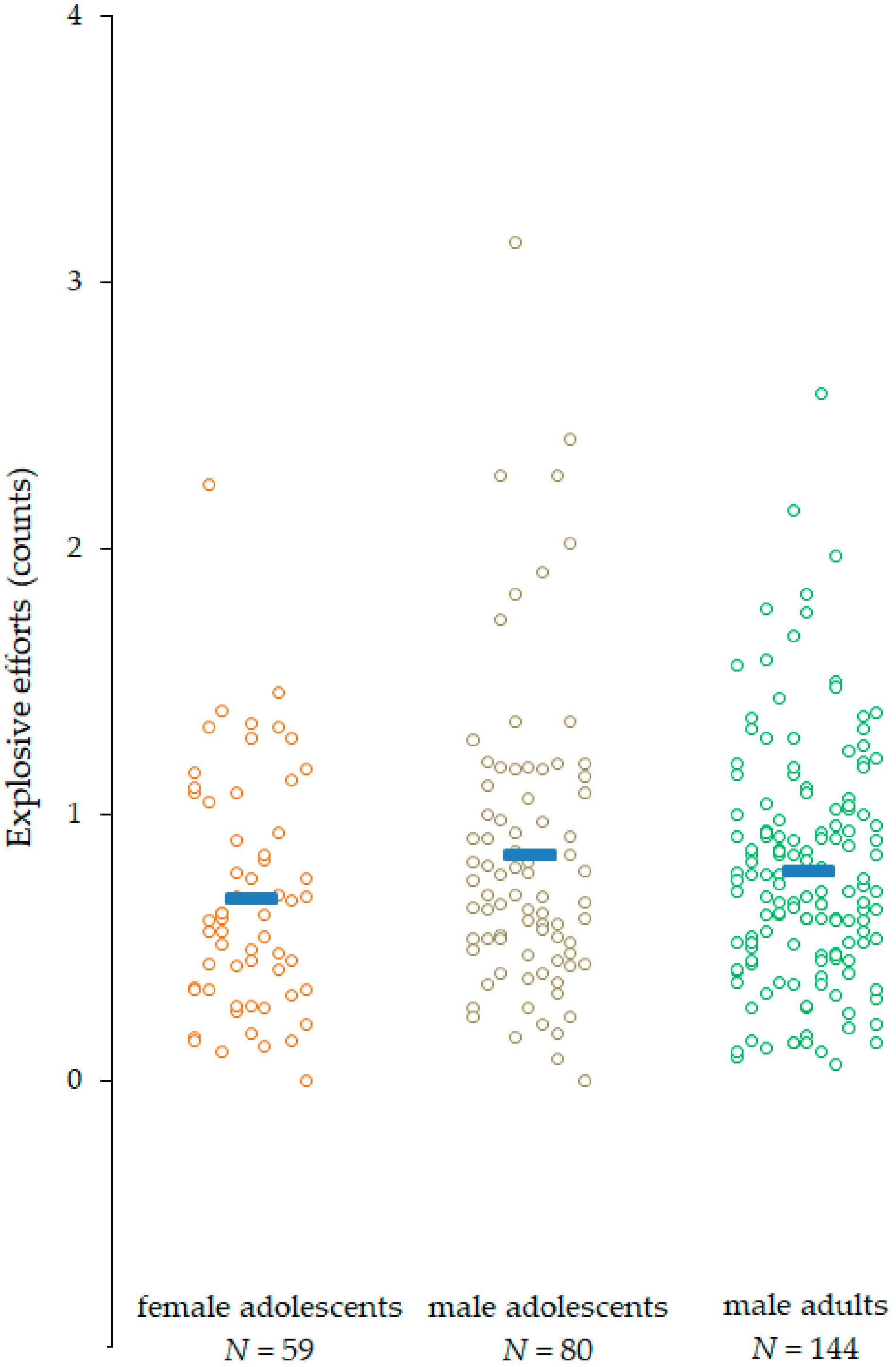

Explosive efforts (counts)
4
3
2
1
0
female adolescents
N = 59
male adolescents
N = 80
male adults
N = 144

(**a**)

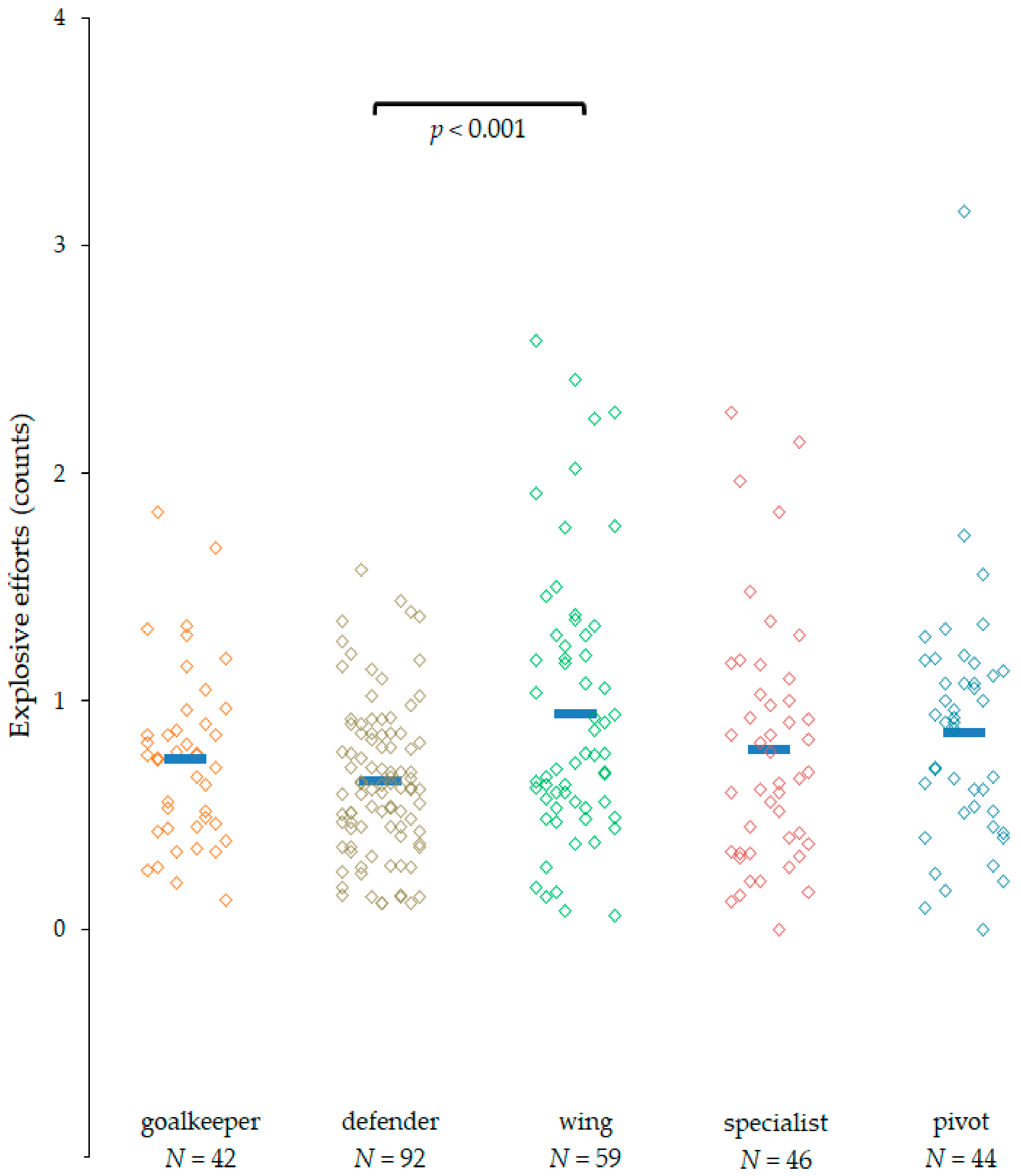

(**b**)

Figure S5. Explosive efforts per minute (counts) across the subgroups: (**a**) subgroup 1 (age/sex); (**b**) subgroup 2 (playing position).

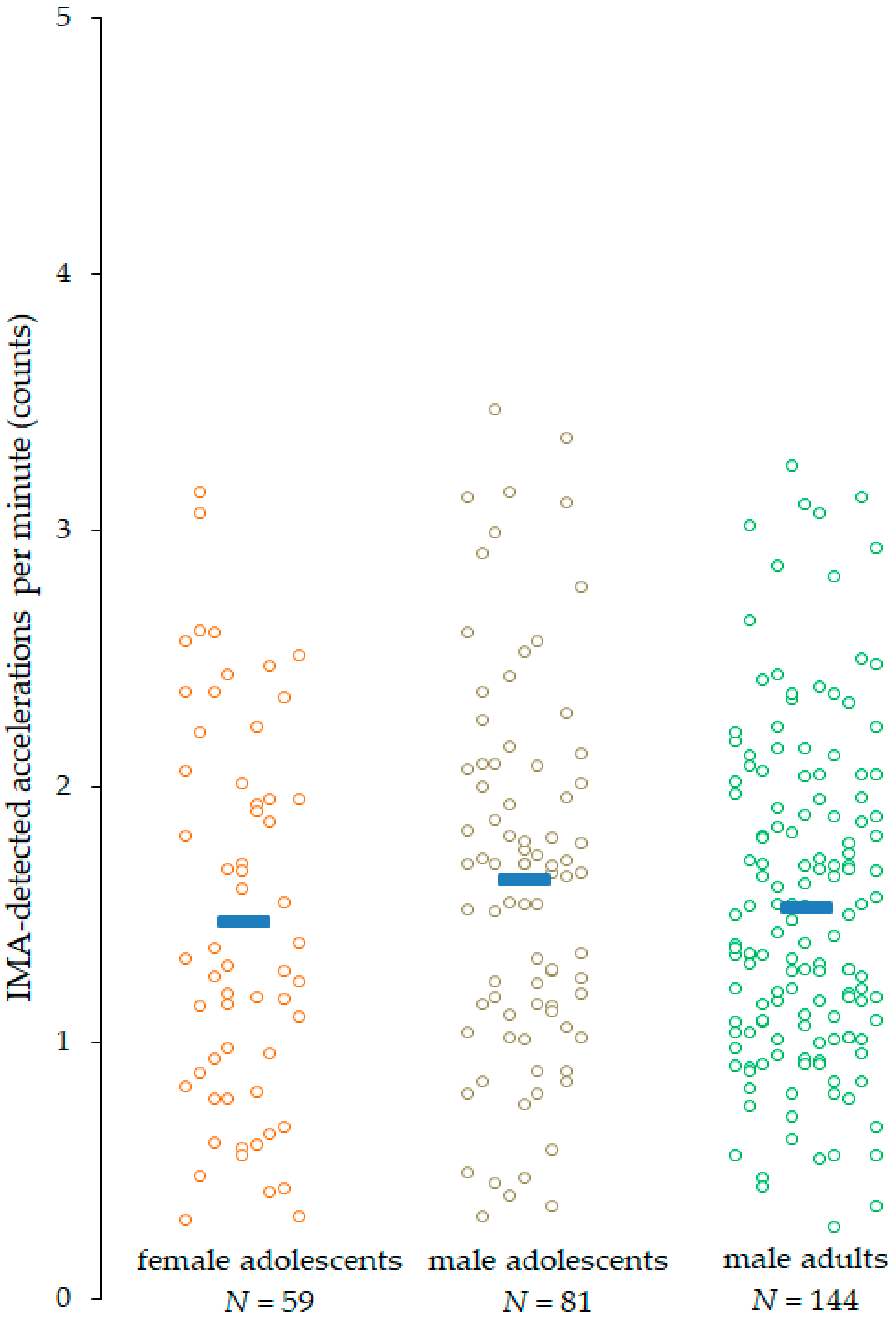

IMA-detected accelerations per minute (counts)
5
4
3
2
1
0
female adolescents
N = 59
male adolescents
N = 81
male adults
N = 144

(a)

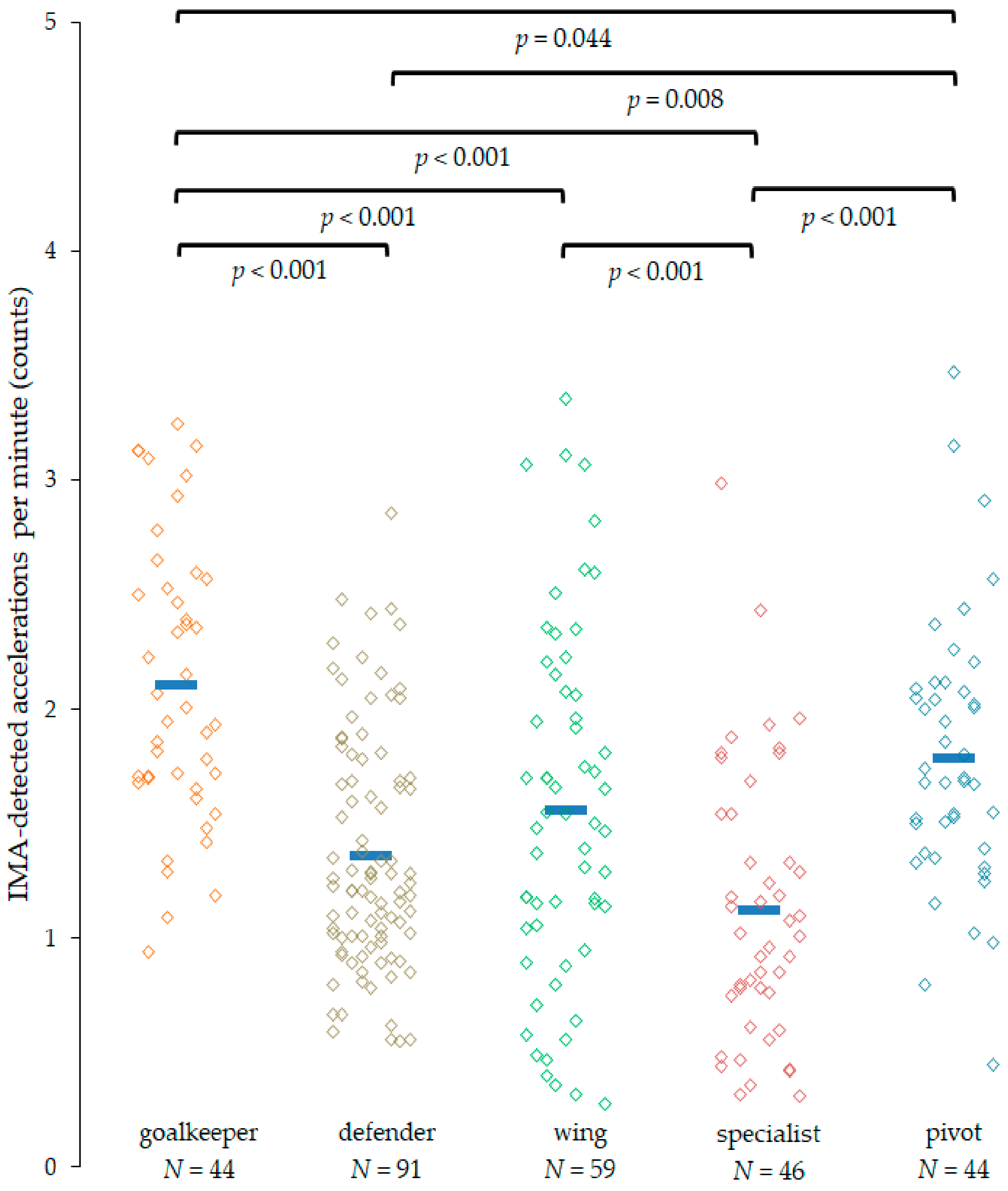

(**b**)

Figure S6. IMA-detected accelerations per minute (counts) across the subgroups: (**a**) subgroup 1 (age/sex); (**b**) subgroup 2 (playing position).

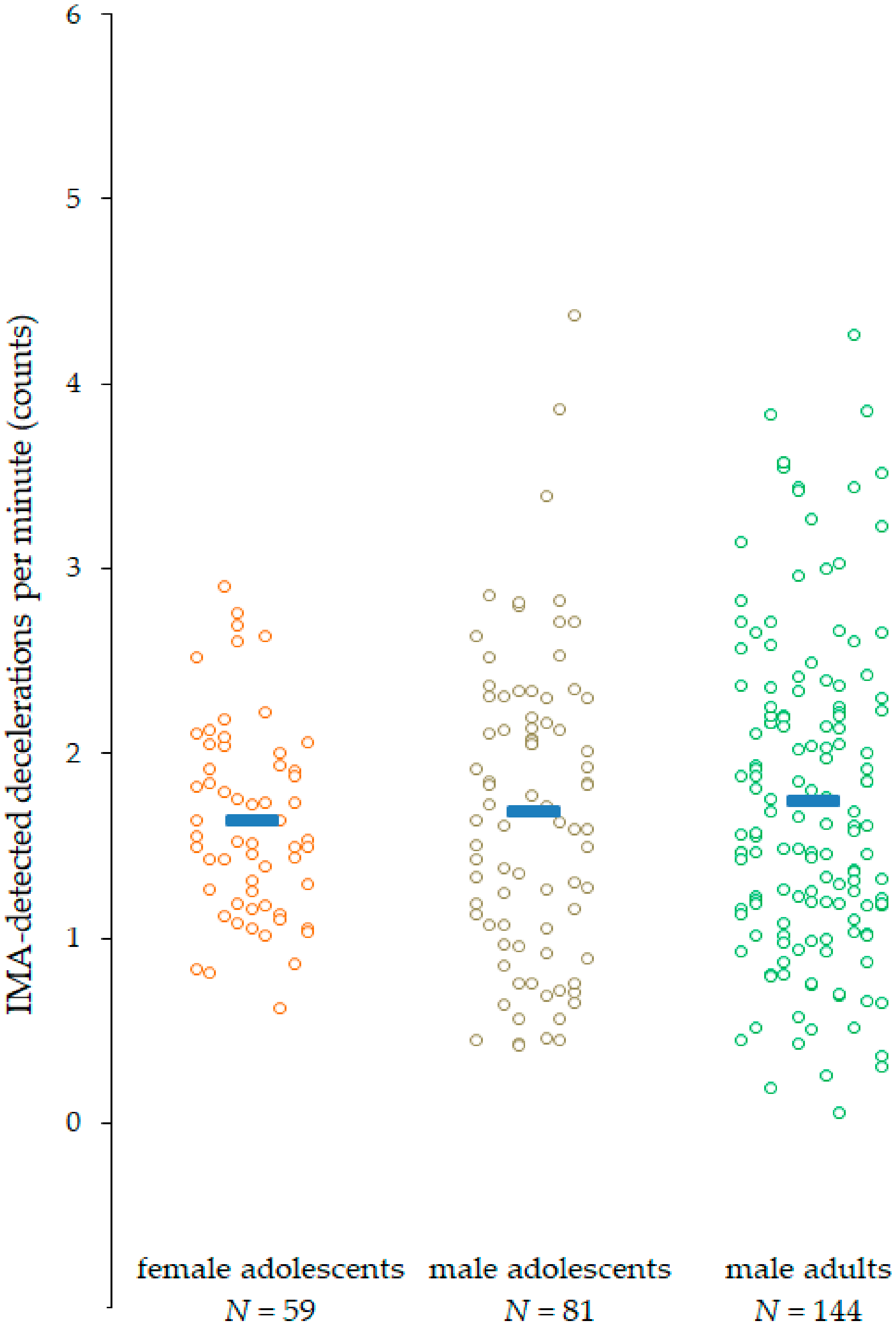

IMA-detected decelerations per minute (counts)
0
1
2
3
4
5
6
female adolescents
N = 59
male adolescents
N = 81
male adults
N = 144

(**a**)

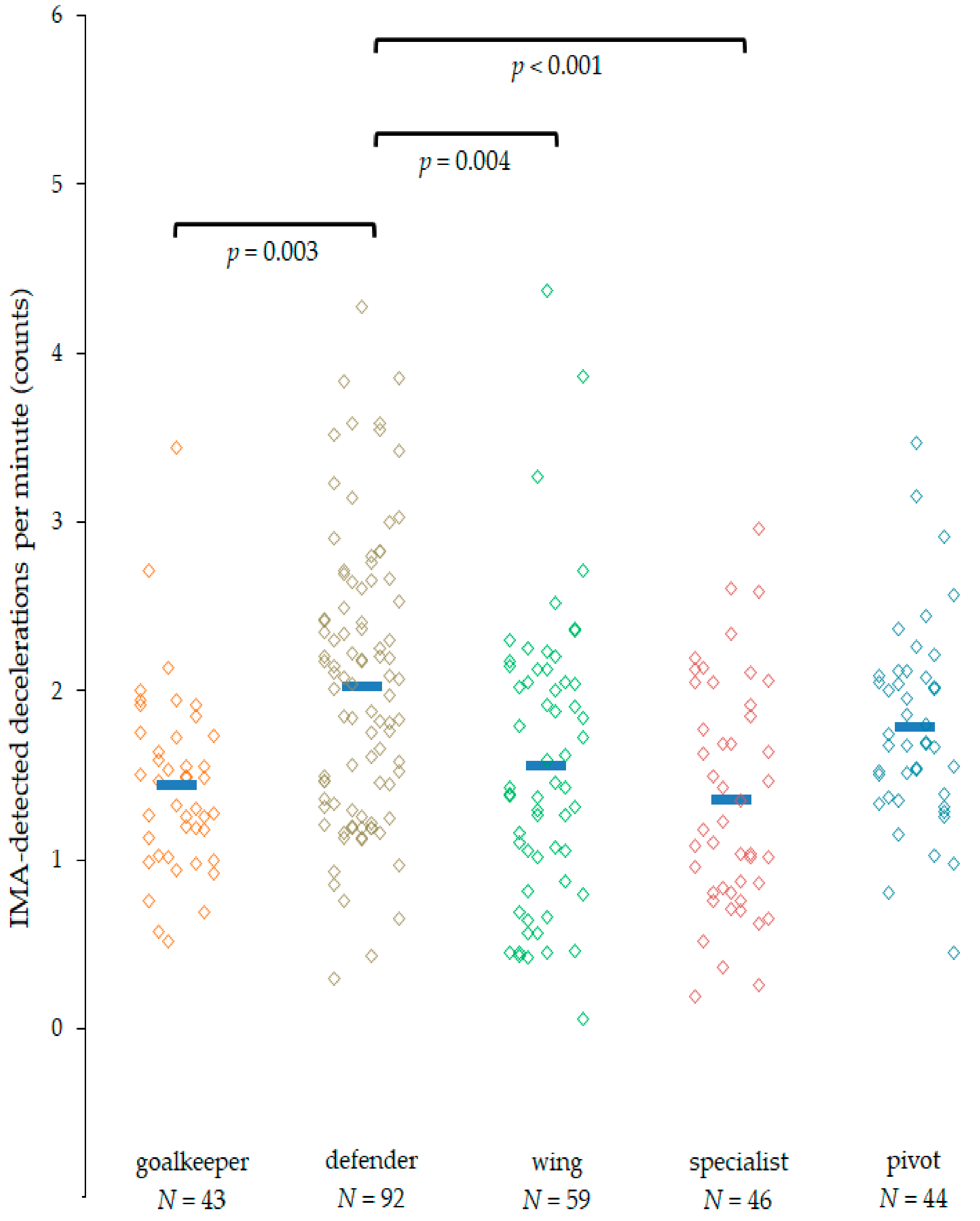

(**b**)

Figure S7. IMA-detected decelerations per minute (counts) across the subgroups: (**a**) subgroup 1 (age/sex); (**b**) subgroup 2 (playing position).

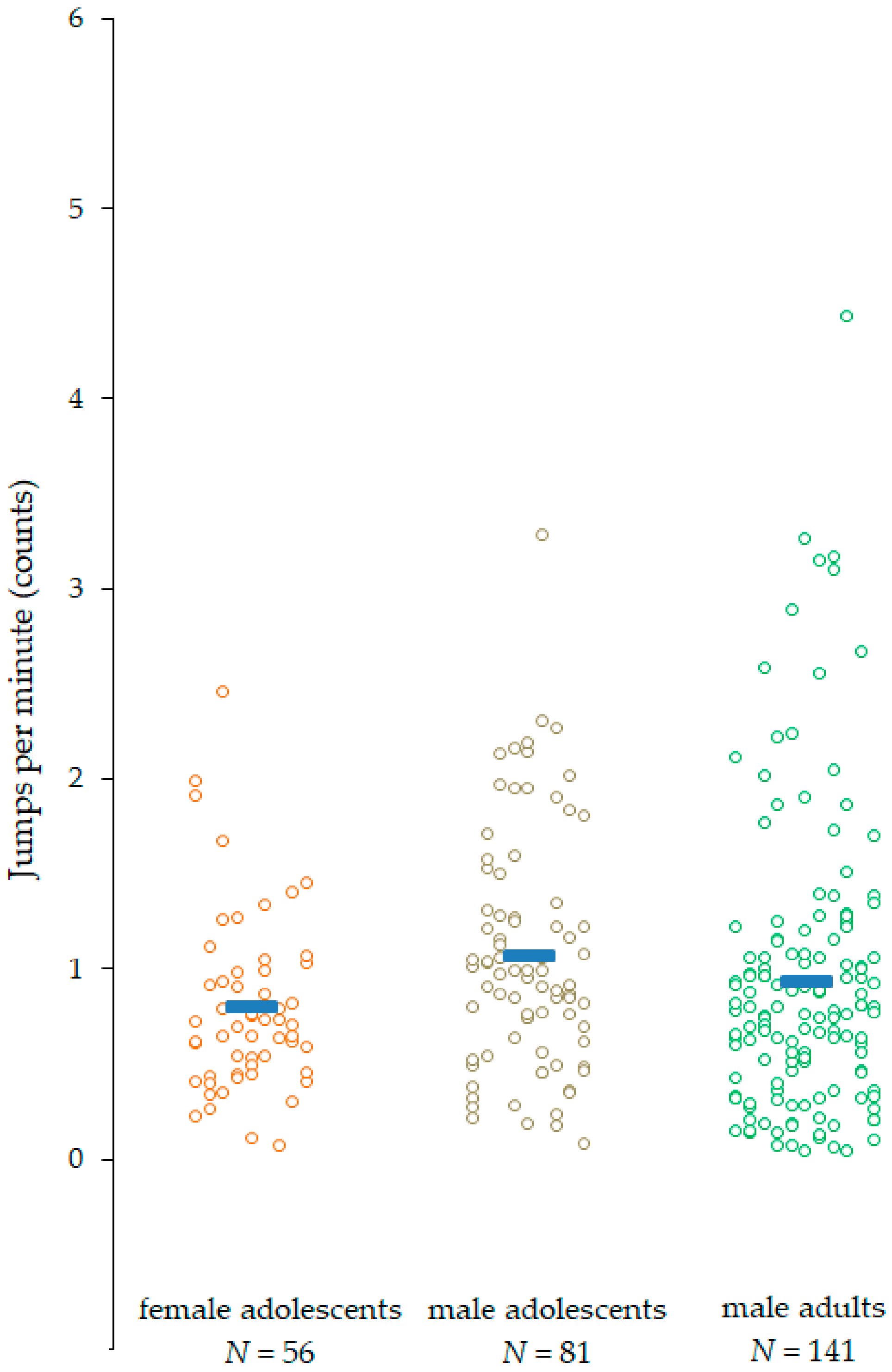

6
5
4
3
2
1
0
Jumps per minute (counts)
female adolescents
N = 56
male adolescents
N = 81
male adults
N = 141

(a)

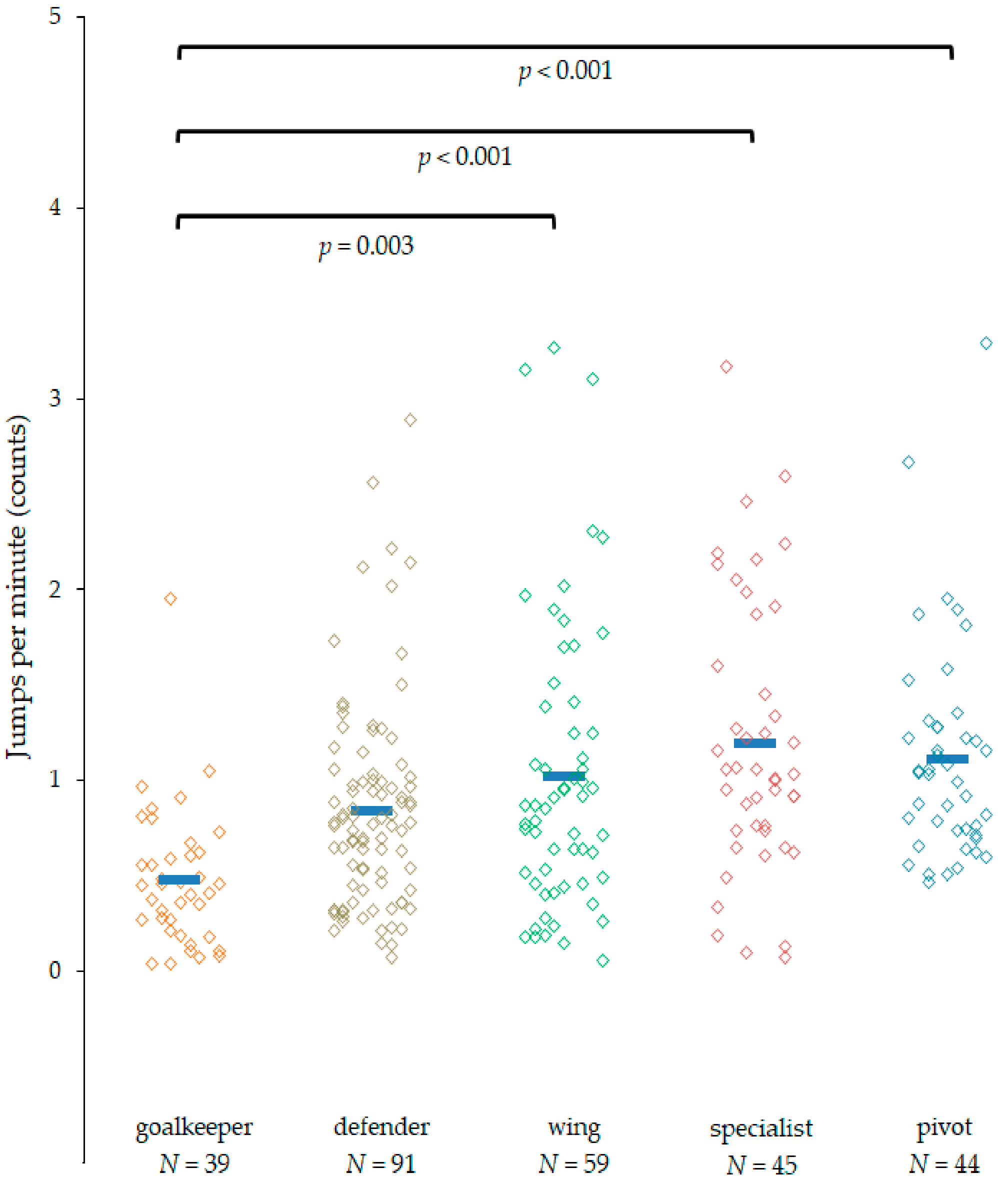

(**b**)

Figure S8. Jumps per minute (counts) across the subgroups: (**a**) subgroup 1 (age/sex); (**b**) subgroup 2 (playing position).
